# Global Epidemiological Patterns in the Burden of Main Non-Communicable Diseases, 1990–2019: Relationships With Socio-Demographic Index

**DOI:** 10.3389/ijph.2023.1605502

**Published:** 2023-01-16

**Authors:** Jianjun Bai, Jiaxin Cui, Fang Shi, Chuanhua Yu

**Affiliations:** ^1^ School of Public Health, Wuhan University, Wuhan, China; ^2^ School of Nursing, Wuhan University, Wuhan, China

**Keywords:** cardiovascular diseases, Global Burden of Disease, neoplasms, chronic respiratory diseases, socio-demographic index

## Abstract

**Objectives:** This study aimed to analyze spatio-temporal patterns of the global burden caused by main NCDs along the socio-economic development.

**Methods:** We extracted relevant data from GBD 2019. The estimated annual percentage changes, quantile regression and limited cubic splines were adopted to estimate temporal trends and relationships with socio-demographic index.

**Results:** NCDs accounted for 74.36% of global all-cause deaths in 2019. The main NCDs diseases were estimated for cardiovascular diseases, neoplasms, and chronic respiratory diseases, with deaths of 18.56 (17.08–19.72) million, 10.08 (9.41–10.66) million and 3.97 (3.58–4.30) million, respectively. The death burden of three diseases gradually decreased globally over time. Regional and sex variations existed worldwide. Besides, the death burden of CVD showed the inverted U-shaped associations with SDI, while neoplasms were positively correlated with SDI, and CRD showed the negative association.

**Conclusion:** NCDs remain a crucial public health issue worldwide, though several favorable trends of CVD, neoplasms and CRD were observed. Regional and sex disparities still existed. Public health managers should execute more targeted programs to lessen NCDs burden, predominantly among lower SDI countries.

## Introduction

Over the last 3 decades, the global health landscape has undergone tremendous transformation along with economic expansion, population swell, aging, greater longevity, unhealthy lifestyle and environmental pollution, which collectively lead to marked changes in the pattern of disease burden [1, 2]. Epidemiologic evidence suggests that non-communicable diseases (NCDs) have been the main contributors to the mortality and disability adjusted life years (DALY) worldwide [3, 4]. Meanwhile, these NCDs are no longer diseases of affluence, but affect all countries at different socio-economic levels [5].

The underlying reasons for this transformation may be the population aging and the rapid decline in deaths from communicable, maternal, neonatal, and nutritional causes (CMNN) [3]. During the past decades, many programs, like Millennium Development Goals (MDG), have significantly reduced the deaths from CMNN, including lower respiratory infections, measles, neonatal disorders, tuberculosis and so on [6, 7]. According to GBD 2017, CMNN accounted for 18.6% of global deaths, with the number of CMNN deaths decreasing by 22.2% and mortality decreasing by 31.8% since 2007. Whereas NCDs have already made up the most important part, accounting for 73.4% of global deaths. Deaths from NCDs increased worldwide, from 33.5 million in 2007 to 41.1 million in 2017. The most enormous numbers of deaths from NCDs were estimated for cardiovascular diseases (CVD, 17.8 million), followed by neoplasms (9.56 million) and chronic respiratory diseases (CRD, 3.91 million) [8]. Population aging and growth may amplify the speed of this shift from CMNN to NCDs [9].

NCDs are not just one of the most pressing global health concerns, but also a significant development challenge and human rights issue [3]. Given this apparent shift and the increasing NCD burden worldwide, the United Nations released the Sustainable Development Goals (SDG) target 3.4 to reduce the premature mortality from NCDs by a third by 2030 from several aspects, such as poverty, economic growth, education, and gender disparity [10]. Meanwhile, many developing countries, like Brazil, Russia, India, China, and South Africa, also affirmed to make efforts to achieve the SDGs 2030 [11]. Hence, to judge the gap between current conditions and SDGs, it is essential to explore the spatial distribution disparities of the burden caused by main NCDs, and evaluate the secular trends over the recent period on a global scale, especially among countries or regions with different social-economic levels.

In this study, we analyzed the spatio-temporal patterns of the burden caused by CVD, neoplasms, and CRD at national, regional, and global scales and assessed the association between socio-demographic index (SDI) and burden of disease.

## Methods

### Data Sources

The Global Burden of Diseases (GBD) 2019 provided a systematic and comprehensive annual assessment of 369 diseases and injuries, 87 behavioral, environmental, occupational, and metabolic risk factors among 204 countries or territories, and 21 GBD regions over the past 30 years. Detailed information can be found in the Global Health Data Exchange (GHDx) query tool (http://ghdx.healthdata.org/gbd-results-tool). As a secondary study, we extracted data on annual deaths, DALYs, mortality, DALY rates, age standardized mortality rates (ASMRs) and age standardized DALY rates (ASDRs) for CVD, neoplasms, and CRD from 1990 to 2019. The SDI is used to evaluate the social development level comprehensively. It is a composite indicator of income *per capita*, average education for those aged 15 and older, and total fertility rate under 25, ranging from 0 to 1, i.e., least to most developed ones [12]. Based on SDI, the 204 countries and territories are divided into five regions: low (less than 0.46), low-middle (0.46–0.60), middle (0.61–0.69), high-middle (0.70–0.81), and high (greater than 0.81) SDI regions.

The detailed methodology of GBD 2019 has been described previously [13, 14]. We have provided the ICD-10 codes for CVD, neoplasms and CRD in Supplementary Table S1. Briefly, 86,249 sources were included in GBD 2019 analysis, including censuses, household surveys, civil registration and vital statistics, disease registries, systematic literature search and so on. To ensure comparability of multiple data sources, the GBD utilized standardized data identification, extraction, and processing methods to address data incompleteness, discrepancies in coding practices, and inconsistent sex and age group reports [15]. A standard cause of death ensemble model (CODEm) and DisMod-MR2.1 synthesized all available data sources to produce internally consistent mortality and prevalence estimates. Years of life lost (YLLs) were calculated by using global standard life expectancy and death counts by age based on the GBD 2019 reference life table [13, 16]. Years lived with disability (YLDs) were determined by multiplying sequela-specific prevalence by the corresponding disability weights. DALYs were the sum of YLLs and YLDs. And GBD generated 95% uncertainty intervals (UI) for all reported data based on the 25th and 975th ordered values of 1,000 draws of the posterior distribution [17].

### Statistical Analysis

We used the age standardized rates (ASRs) and estimated annual percentage change (EAPC) to quantify the trends of disease burden. ASRs were required when comparing several different populations or the same population across time with different age structures. Besides, the ASR trends could serve as good proxies to evaluate the transformation of disease patterns and the efficacy of current preventative initiatives.

$$ASR=\frac{\sum_{i=1}^{A}a_{i}w_{i}}{\overset{A}{\underset{i=1}{\sum }}w_{i}}$$

where $a_{i}$ was the age-specific rate of *i*th age group, $w_{i}$ was the number of persons in the same *i*th age group of the reference standard population. ASRs were based on the GBD 2019 global standard population.

The EAPC has been widely used in previous studies [18–20], reflecting the trend of ASRs over time. A regression line was fitted to the natural logarithm of the ASRs:

$$\ln\left(ASR\right)=a+bx+e$$

x=calendar year

$$EAPC=100*\left(\exp\left(b\right)-1\right)$$

EAPCs and its 95% confidence interval (CI) can be obtained from the linear regression model. If the estimated EAPC and the lower bound of its 95% CI were both >0, then ASR was considered to show the upward trend; if both the estimated EAPC and the upper bound of its 95% CI were <0, then ASR was on the downward trend; otherwise, ASR was considered to be stable.

We also applied the restricted cubic splines (RCS) with the lowest Akaike information criterion to explore the relationship between SDI and ASMRs, ASDRs, gender differences of ASRs (males’-females’), YLD-YLL ratios, EAPCs. The YLD-YLL ratios were calculated by dividing the age standardized YLD rate by the age standardized YLL rate to reflect the change in YLDs proportion. RCS is essentially the regression splines with added constraints. The regression spline is a piecewise polynomial in which each piece must have a continuous and second-order derivative. Regression splines are required for restricted cubic splines: the spline function is a linear function at the extreme ends of the independent variable data range [21].

Quantile regression (QR) was further applied to determine whether the influence of SDI on target disease burden varied across different quantiles. The change in disease burden due to SDI in specific percentiles has depicted in Supplementary Table S2. QR estimates the linear relationship between the different quantiles of the dependent and independent variables and attempts to comprehensively present all data information, which is an advantage over the traditional linear regression model [21]. The estimation of QR is based on the minimum weighted absolute value residual [22]. The minimum weighted absolute deviation of quantile regression is as follows:

$$\min\left\{w_{t}\left|y_{t}-\alpha \right|\right\}=-\overset{T}{\underset{i:y_{i}<\alpha }{\sum }}\left(1-\tau \right)\left(y_{t}-\alpha \right)+\overset{T}{\underset{i:y_{i}\geq \alpha }{\sum }}\tau \left(y_{t}-\alpha \right)$$

ArcGIS (10.5) was used for mapping to reflect the geographic variability of the target disease burden. Statistical analyses were performed using Python software (3.7) and R software (4.0.2). Two-sided *p* < 0.05 was considered statistically significant.

## Results

### Transmission of Disease Burden and SDI

The global number of deaths from NCDs was 42.03 million (95% UI 40.08–43.94) in 2019, which accounted for 74.36% of all-cause deaths. The most enormous numbers of deaths were estimated for cardiovascular diseases (18.56 million [17.08–19.72]), followed by neoplasms (10.08 million [9.41–10.66]) and chronic respiratory diseases (3.97 million [3.58–4.30]). Among 204 countries or territories, the highest SDI was observed in Switzerland (0.929), followed by Norway (0.913) and Monaco (0.902). The SDI of Niger (0.162), Chad (0.238), and Burkina Faso (0.257) were the lowest. For 21 GBD regions, the SDI in High-income Asia Pacific (0.873) was the highest, followed by High-income North America (0.860) and Western Europe (0.843) (Supplementary Figure S1). Compared with the SDI in 1990, the countries with obvious increases in SDI were mainly concentrated in Africa, like Equatorial Guinea (229.33%), Mozambique (155.83%), and Uganda (141.92%) (Supplementary Figure S1).

### Disease Burden Due to Cardiovascular Diseases

**FIGURE 1:**
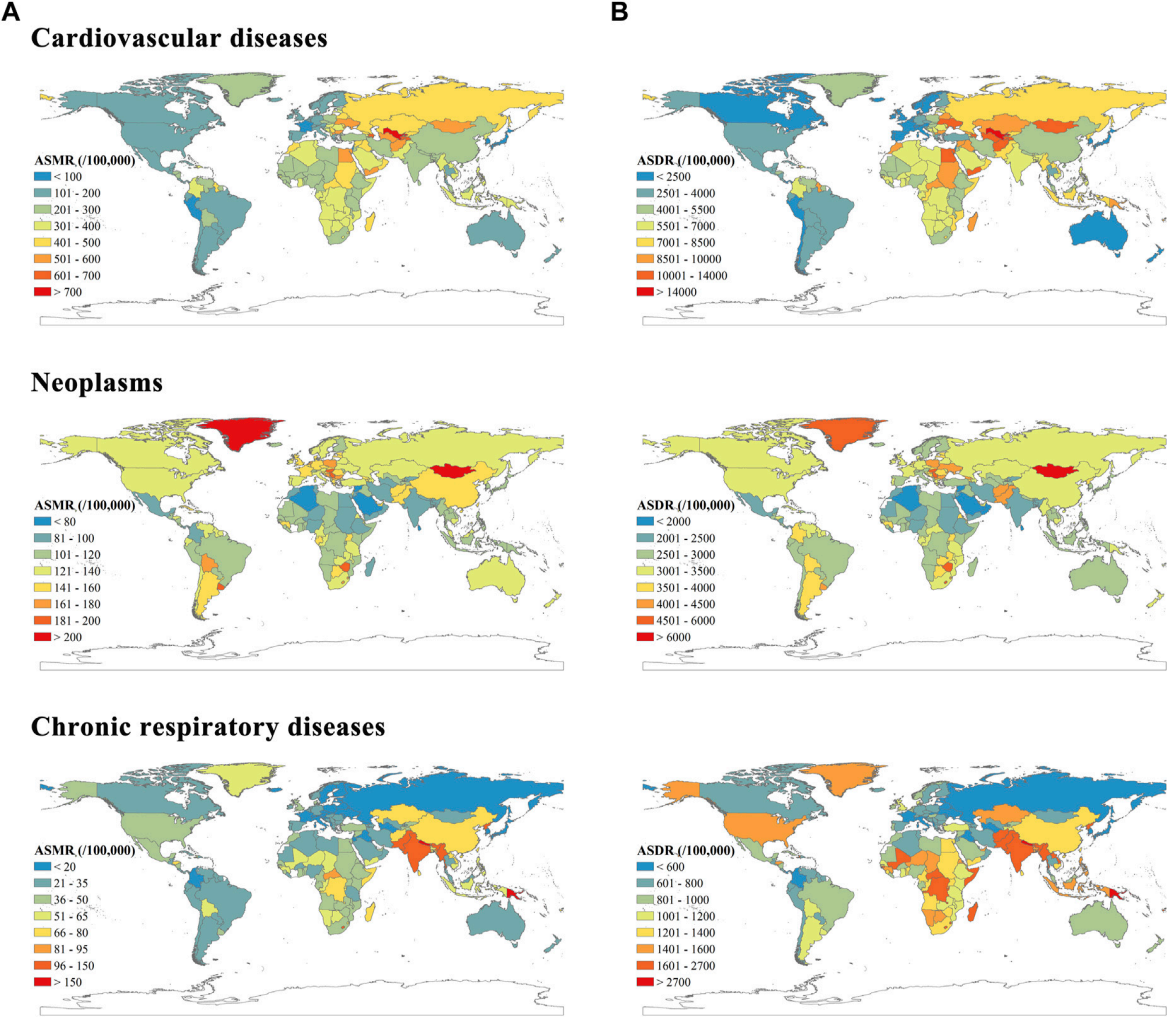
Age standardized mortality rates **(A)** and age standardized Disability adjusted life years rates **(B)** per 100,000 people of cardiovascular diseases, neoplasms and chronic respiratory diseases. Global Burden of Diseases Study, 204 countries or territories, 2019.

According to GBD 2019, the ASMRs of CVD varied considerably across the world (Figure 1). Four of six countries with ASMRs below 100 per 100,000 were high SDI countries (Japan, South Korea, Singapore, and France). And the fastest decrease in ASMR has been seen in South Korea, with the EAPC of −5.19 (95% CI −5.39 to −4.98) (Supplementary Figure S2). The ASDRs of CVD were significantly heterogeneous globally, with the highest ASDR observed in the Solomon Islands (20,181.50; 16,678.86–23,957.75) and the lowest in Japan (1,620.47; 1,460.11–1744.70) (Figure 1). Besides, 14 of 16 countries with ASDRs exceeding 10,000 per 100,000 were from low, low-middle, and middle SDI regions.

Table 1 showed ASMRs and ASDRs of CVD in 1990 and 2019 and corresponding temporal trends. The ASMR and ASDR decreased across the world and all five SDI regions from 1990 to 2019. The most rapid declines for both ASMR and ASDR were detected in the high SDI region, with EAPCs of −2.86 (95% CI −3.00 to −2.72) and −2.63 (−2.77 to −2.49), respectively. While in recent years, the declines have stagnated in the high SDI region (Supplementary Figure S3). Among 21 geographical regions, the most significant decrease in ASMR was observed in High-income Asia Pacific (EAPC = −3.54, −3.73 to −3.35), followed by Australasia and Western Europe. Meanwhile, the most rapid decrease in ASDR was detected in Australasia (EAPC = −3.56, −3.76 to −3.35), followed by High-income Asia Pacific and Western Europe. All these were from high SDI region.

**TABLE 1:** The age standardized mortality rate (A) and age standardized Disability adjusted life years rate (B) and corresponding temporal trends of cardiovascular diseases. Global Burden of Diseases Study, 21 Global Burden of Diseases regions, 1990–2019.

A	Age standardized mortality rates (95% UI)		EAPC (95% CI) 1990–2019
	1990	2019	
Global	354.50 (330.59–369.49)	239.85 (219.43–254.90)	−1.46 (−1.51 to −1.40)
Sex			
Male	401.95 (380.82–418.59)	280.83 (259.16–299.80)	−1.31 (−1.35 to −1.26)
Female	314.32 (287.8–332.97)	204.03 (180.93–221.55)	−1.63 (−1.70 to −1.57)
SDI			
Low	339.69 (303.35–380.09)	284.08 (257.12–310.93)	−0.67 (−0.71 to −0.62)
Low-middle	341.41 (314.53–368.89)	284.43 (259.26–308.41)	−0.65 (−0.70 to −0.61)
Middle	367.21 (339.93–392.16)	281.76 (255.69–304.53)	−0.82 (−0.89 to −0.76)
High-middle	423.47 (394.78–439.79)	262.74 (237.98–280.34)	−1.91 (−2.09 to −1.73)
High	274.36 (255.06–284.04)	129.43 (114.72–137.20)	−2.86 (−3.00 to −2.72)
GBD region			
Andean Latin America	213.78 (191.60–233.90)	123.99 (102.72–147.53)	−1.82 (−2.03 to −1.60)
Australasia	280.08 (257.77–290.53)	112.78 (98.11–120.87)	−3.51 (−3.69 to −3.33)
Caribbean	330.21 (308.22–347.56)	241.83 (211.00–273.98)	−1.06 (−1.24 to −0.89)
Central Asia	517.84 (487.70–534.76)	575.23 (531.07–621.33)	0.08 (−0.33 to 0.48)
Central Europe	531.11 (500.99–544.77)	310.38 (272.62–346.98)	−2.20 (−2.32 to −2.07)
Central Latin America	233.14 (215.97–241.71)	161.18 (140.07–184.69)	−1.45 (−1.60 to −1.30)
Central Sub-Saharan Africa	373.82 (324.72–427.14)	321.50 (268.61–384.43)	−0.57 (−0.62 to −0.51)
East Asia	377.77 (338.68–421.66)	273.22 (237.70–307.56)	−0.92 (−1.03 to −0.80)
Eastern Europe	558.43 (529.67–573.09)	464.70 (416.21–506.84)	−1.15 (−1.59 to −0.70)
Eastern Sub-Saharan Africa	323.99 (288.01–357.74)	270.69 (241.55–298.16)	−0.69 (−0.72 to −0.66)
High-income Asia Pacific	215.59 (196.89–224.12)	79.11 (66.12–86.58)	−3.54 (−3.73 to −3.35)
High-income North America	261.95 (242.41–271.35)	151.69 (136.72–159.59)	−2.22 (−2.38 to −2.07)
North Africa and Middle East	498.52 (459.71–530.96)	358.87 (320.58–394.18)	−1.19 (−1.24 to −1.14)
Oceania	407.04 (345.26–478.96)	401.28 (332.77–483.90)	0.00 (−0.04 to 0.03)
South Asia	332.14 (298.37–367.22)	266.86 (233.51–298.95)	−0.85 (−0.96 to −0.75)
Southeast Asia	324.64 (296.61–349.63)	289.93 (260.52–313.80)	−0.29 (−0.39 to −0.18)
Southern Latin America	316.99 (297.24–327.13)	166.25 (152.05–175.01)	−2.26 (−2.43 to −2.10)
Southern Sub-Saharan Africa	243.27 (220.77–262.89)	243.87 (222.52–262.07)	0.04 (–0.37 to 0.44)
Tropical Latin America	352.07 (329.35–364.48)	175.92 (158.90–185.22)	−2.41 (−2.50 to −2.31)
Western Europe	284.35 (264.61–294.31)	128.05 (113.02–135.38)	−3.04 (−3.19 to −2.89)
Western Sub-Saharan Africa	312.47 (266.40–374.67)	260.87 (224.99–294.84)	−0.66 (−0.73 to −0.60)

B	Age Standardized DALY Rates (95% UI)		EAPC (95% CI) 1990–2019
	1990	2019	
Global	7,084.63 (6,766.20–7,375.97)	4,863.64 (4,548.71–5,164.24)	−1.40 (−1.45 to −1.35)
Sex			
Male	8,252.53 (7,883.51–8,596.74)	5,851.28 (5,448.15–6,247.67)	−1.25 (−1.30 to −1.20)
Female	6,025.26 (5,651.26–6,361.67)	3,948.54 (3,616.57–4,248.62)	−1.60 (−1.67 to −1.54)
SDI			
Low	7,534.15 (6,820.08–8,269.70)	6,077.79 (5,526.59–6,696.56)	−0.80 (−0.85 to −0.76)
Low-middle	7,403.56 (6,911.57–7,920.40)	6,031.21 (5,513.27–6,554.67)	−0.71 (−0.75 to −0.67)
Middle	7,466.84 (7,001.58–7,934.96)	5,488.14 (5,077.63–5,894.26)	−0.99 (−1.03 to −0.94)
High-middle	7,954.02 (7,594.62–8,257.17)	4,890.90 (4,538.73–5,189.41)	−1.98 (−2.19 to −1.76)
High	5,100.77 (4,879.30–5,266.77)	2,531.30 (2,366.77–2,676.66)	−2.63 (−2.77 to −2.49)
GBD region			
Andean Latin America	4,407.04 (4,008.64–4,800.91)	2,464.29 (2070.01–2,920.30)	−1.99 (−2.19 to −1.78)
Australasia	5,020.87 (4,769.29–5,189.52)	1951.20 (1800.15–2085.34)	−3.56 (−3.76 to −3.35)
Caribbean	6,870.87 (6,529.51–7,238.32)	5,171.80 (4,496.01–5,907.59)	−0.96 (−1.14 to −0.78)
Central Asia	10,284.06 (9,928.74–10,562.31)	10,664.54 (9,836.53–11,621.47)	−0.22 (−0.63 to 0.18)
Central Europe	9,823.5 (9,466.78–10,056.44)	5,391.06 (4,768.40–6,047.76)	−2.45 (−2.58 to −2.32)
Central Latin America	4,547.69 (4,347.33–4,681.83)	3,110.43 (2,720.37–3,570.10)	−1.49 (−1.63 to −1.34)
Central Sub-Saharan Africa	8,071.58 (6,992.56–9,265.43)	6,561.71 (5,441.43–7,946.19)	−0.77 (−0.82 to −0.71)
East Asia	7,368.39 (6,628.00–8,178.42)	4,915.14 (4,312.64–5,528.93)	−1.26 (−1.34 to −1.18)
Eastern Europe	10,325.06 (10,007.14–10,552.09)	9,002.53 (8,171.25–9,868.45)	−1.02 (−1.55 to −0.50)
Eastern Sub-Saharan Africa	7,187.17 (6,413.12–7,962.09)	5,577.55 (4,995.13–6,171.11)	−0.98 (−1.02 to −0.93)
High-income Asia Pacific	3,945.77 (3,724.18–4,098.05)	1,605.38 (1,450.10–1728.99)	−3.16 (−3.31 to −3.01)
High-income North America	5,160.97 (4,925.25–5,342.20)	3,099.00 (2,912.68–3,259.12)	−2.00 (−2.13 to −1.86)
North Africa and Middle East	10,508.11 (9,866.12–11,179.87)	7,089.12 (6,309.75–7,858.00)	−1.45 (−1.49 to −1.40)
Oceania	9,901.00 (8,390.14–11,630.13)	9,681.75 (7,933.36–11,767.00)	−0.01 (−0.06 to 0.04)
South Asia	7,250.61 (6,604.49–7,873.90)	5,798.52 (5,142.95–6,491.30)	−0.81 (−0.89 to −0.74)
Southeast Asia	7,016.99 (6,508.80–7,499.09)	6,121.89 (5,562.06–6,619.15)	−0.36 (−0.45 to −0.27)
Southern Latin America	6,112.05 (5,903.28–6,267.94)	3,144.46 (2,974.97–3,292.74)	−2.38 (−2.54 to −2.23)
Southern Sub-Saharan Africa	5,300.98 (4,905.07–5,665.42)	4,802.63 (4,422.15–5,182.61)	−0.31 (−0.70 to 0.08)
Tropical Latin America	7,430.07 (7,148.41–7,633.93)	3,768.91 (3,564.06–3,937.96)	−2.38 (−2.45 to −2.31)
Western Europe	4,975.32 (4,753.27–5,129.66)	2,158.15 (2001.70–2,274.85)	−3.16 (−3.31 to −3.00)
Western Sub-Saharan Africa	6,476.48 (5,514.41–7,725.67)	5,278.91 (4,573.20–6,022.66)	−0.75 (−0.83 to −0.68)

**FIGURE 2:**
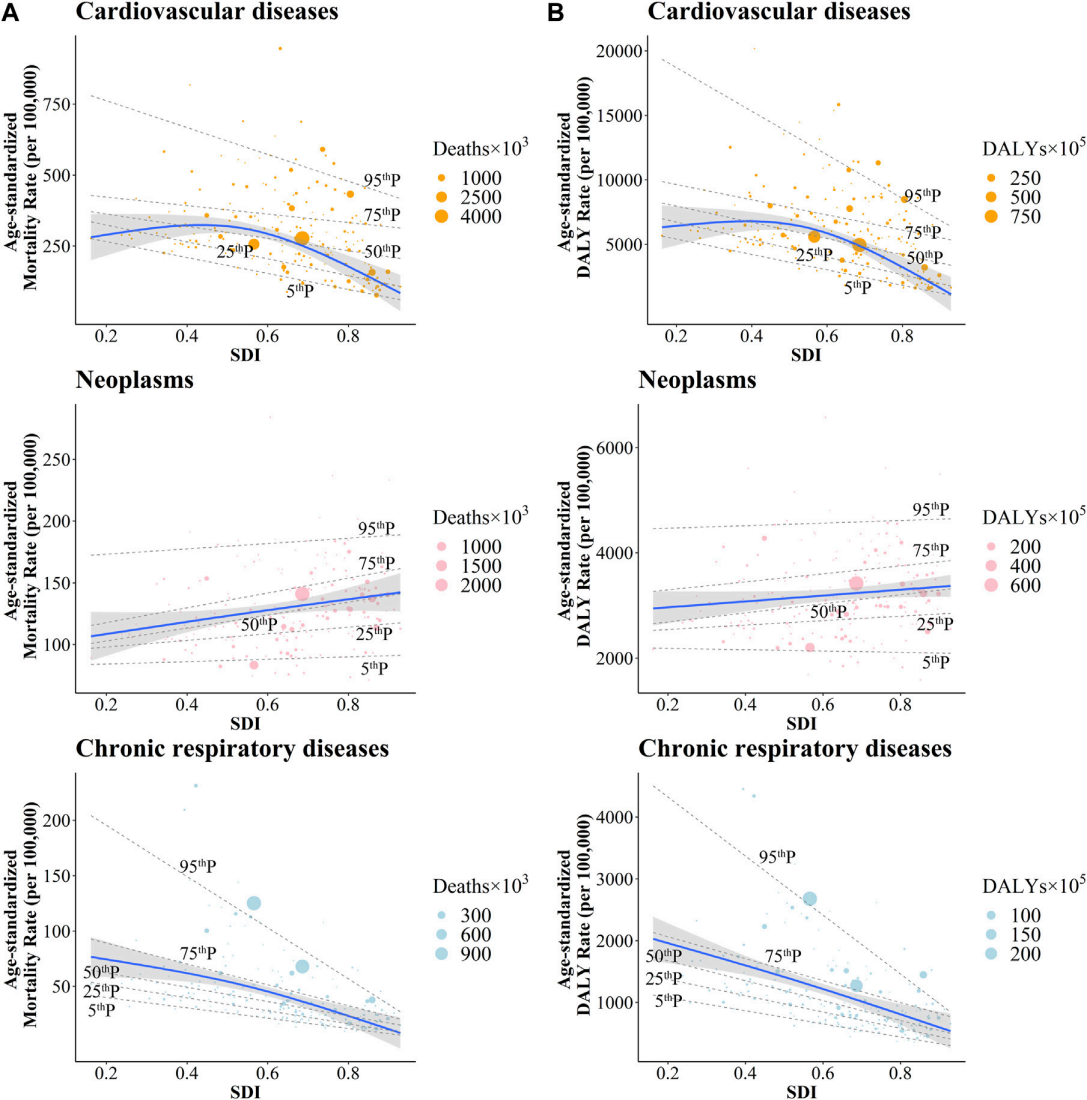
The correlation between the socio-demographic index with age standardized mortality rates **(A)** and age standardized Disability adjusted life years rates **(B)** of cardiovascular diseases, neoplasms and chronic respiratory diseases. Global Burden of Diseases Study, 204 countries or territories, 2019.

**FIGURE 3:**
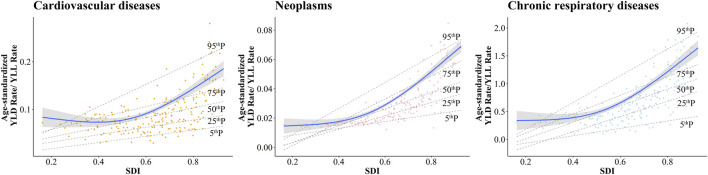
The correlation between the socio-demographic index and the ratios of Years lived with disability—Years of life lost for cardiovascular diseases, neoplasms and chronic respiratory diseases. Global Burden of Diseases Study, 204 countries or territories, 2019.

The estimated association between SDI and ASRs due to CVD were showed as the blue line in Figure 2. Based on the restricted cubic spline, the trends in ASMR and ASDR were increasing first and then decreasing, with inflection points of 0.55 and 0.52, respectively. The overall positive association were observed between SDI and YLD-YLL ratio (Figure 3), suggesting that the proportion of YLD is increasing with SDI. In addition, negative correlations were detected between EAPC and SDI, which means countries with higher SDI have experienced more rapid decreases in ASRs of CVD from 1990 to 2019 (Supplementary Figure S4). Meanwhile, the relationship between the SDI and gender difference of ASMR and ASDR in CVD tended to increase and then decrease with increasing SDI to some extent, but not significantly. Moreover, the burden of CVD was more severe in males than in females, although the decreasing trends of ASMR and ASDR for CVD were observed in each gender (Supplementary Figure S5).

### Disease Burden Due to Neoplasms

As shown in Figure 1, the ASMRs of neoplasms varied considerably, with the highest ASMR in Mongolia (284.33, 95% UI 229.37 to 352.46 per 100,000) and the lowest ASMR in Kuwait (71.26, 60.09–83.80). Similarly, Mongolia showed the highest ASDR (6,578.57, 5,144.24–8,338.00). The lowest ASDR was observed in Kuwait 1,583.93 (1,346.43–1,854.26). The most significant declines in both ASMR and ASDR were observed in Singapore, with EAPCs of −1.95 (−2.03 to −1.87) and −2.23 (−2.30 to −2.16), respectively (Supplementary Figure S2).

Globally, the ASMR decreased from 147.92 (141.18–153.83) to 125.41 (117.07–132.74), with an EAPC of −0.67 (95% CI −0.72 to −0.62). For SDI regions, the declining trends of ASMRs and ASDRs were relatively more obvious in high-middle and high SDI regions (Table 2). For 21 geographical regions, Oceania, Caribbean and Western Sub-Saharan Africa showed increasing trends in ASMRs. And Oceania and Western Sub-Saharan Africa showed increasing trends in ASDRs. The most rapid decreasing trends in ASMR and ASDR were observed in High-income Asia Pacific, with EAPCs of −1.08 (−1.16 to −1.00) and −1.40 (−1.48 to −1.32), respectively (Table 2).

**TABLE 2:** The age standardized mortality rate (A) and age standardized Disability adjusted life years rate (B) and corresponding temporal trends of neoplasms. Global Burden of Diseases Study, 21 Global Burden of Diseases regions, 1990–2019.

A	Age standardized mortality rates (95% UI)		EAPC (95% CI) 1990–2019
	1990	2019	
Global	147.93 (141.18–153.83)	125.41 (117.07–132.74)	−0.67 (−0.72 to −0.62)
Sex			
Male	188.07 (177.09–197.55)	157.14 (144.79–168.27)	−0.70 (−0.75 to −0.65)
Female	118.02 (111.34–124.10)	100.47 (92.010–107.93)	−0.67 (−0.72 to −0.62)
SDI			
Low	101.83 (91.85–111.85)	101.04 (90.33–111.82)	−0.05 (−0.07 to −0.04)
Low-middle	101.55 (94.03–108.65)	102.72 (94.59–111.19)	−0.04 (−0.08 to 0.01)
Middle	136.83 (126.87–146.63)	121.68 (110.42–132.60)	−0.45 (−0.52 to −0.38)
High-middle	164.82 (157.03–171.83)	133.42 (122.10–143.28)	−0.88 (−0.97 to −0.80)
High	166.11 (159.37–169.39)	132.23 (122.62–137.53)	−0.91 (−0.95 to −0.86)
GBD region			
Andean Latin America	130.32 (118.09–143.19)	117.43 (96.91–140.78)	−0.34 (−0.41 to −0.26)
Australasia	164.25 (157.07–168.96)	127.44 (117.19–133.24)	−1.01 (−1.08 to −0.95)
Caribbean	136.89 (127.46–143.10)	135.17 (118.41–154.80)	0.06 (0.00 to 0.13)
Central Asia	141.32 (137.24–144.86)	122.01 (111.58–133.32)	−0.46 (−0.56 to −0.36)
Central Europe	175.14 (170.00–179.83)	163.93 (145.72–184.98)	−0.21 (−0.28 to −0.14)
Central Latin America	114.25 (108.84–118.77)	99.30 (86.06–114.12)	−0.65 (−0.70 to −0.59)
Central Sub-Saharan Africa	120.00 (100.10–141.48)	108.66 (85.47–133.08)	−0.44 (−0.51 to −0.36)
East Asia	172.62 (153.78–190.78)	140.87 (121.47–161.12)	−0.77 (−0.89 to −0.64)
Eastern Europe	156.36 (153.28–159.30)	130.30 (118.19–143.58)	−0.99 (−1.17 to −0.81)
Eastern Sub-Saharan Africa	113.20 (100.67–125.46)	110.49 (96.02–126.99)	−0.13 (−0.16 to −0.10)
High-income Asia Pacific	151.14 (143.95–154.68)	114.65 (102.69–121.48)	−1.08 (−1.16 to −1.00)
High-income North America	168.77 (161.41–172.57)	136.94 (128.98–142.44)	−0.90 (−0.96 to −0.85)
North Africa and Middle East	102.60 (91.66–110.31)	100.23 (90.33–111.04)	0.05 (−0.08 to 0.18)
Oceania	114.05 (97.96–132.57)	123.16 (102.44–150.06)	0.29 (0.27 to 0.32)
South Asia	88.96 (80.17–97.32)	89.24 (79.49–100.27)	−0.12 (−0.20 to −0.04)
Southeast Asia	110.03 (99.65–118.55)	113.00 (100.46–126.99)	−0.02 (−0.06 to 0.03)
Southern Latin America	179.35 (173.15–184.65)	152.21 (144.04–158.81)	−0.66 (−0.72 to −0.60)
Southern Sub-Saharan Africa	132.98 (118.46–149.41)	136.86 (125.55–147.07)	0.01 (−0.26 to 0.29)
Tropical Latin America	132.48 (126.07–140.60)	114.44 (106.52–120.41)	−0.49 (−0.56 to −0.41)
Western Europe	170.90 (164.42–174.23)	137.51 (128.02–142.54)	−0.84 (−0.88 to −0.80)
Western Sub-Saharan Africa	92.06 (80.87–104.28)	106.08 (89.79–122.89)	0.62 (0.57 to 0.67)

B	Age Standardized DALY Rates (95% UI)		EAPC (95% CI) 1990–2019
	1990	2019	
Global	3,823.96 (3,644.86–3,993.15)	3,062.36 (2,871.40–3,237.78)	−0.90 (−0.94 to −0.85)
Sex			
Male	4,602.56 (4,315.13–4,874.08)	3,624.28 (3,341.22–3,891.98)	−0.94 (−0.98 to −0.89)
Female	3,179.90 (3,000.69–3,360.77)	2,583.87 (2,387.31–2,778.41)	−0.86 (−0.92 to −0.81)
SDI			
Low	2,804.51 (2,508.76–3,130.67)	2,663.41 (2,374.49–2,968.24)	−0.21 (−0.24 to −0.19)
Low-middle	2,784.91 (2,570.73–3,007.60)	2,701.18 (2,477.64–2,932.56)	−0.19 (−0.24 to −0.14)
Middle	3,667.67 (3,402.48–3,939.85)	3,004.36 (2,744.11–3,266.04)	−0.77 (−0.82 to −0.72)
High-middle	4,371.98 (4,162.30–4,572.08)	3,267.54 (3,019.83–3,500.78)	−1.22 (−1.30 to −1.13)
High	4,066.15 (3,965.95–4,142.27)	3,035.82 (2,894.39–3,144.78)	−1.14 (−1.18 to −1.10)
GBD region			
Andean Latin America	3,325.37 (3,020.59–3,654.52)	2,833.27 (2,319.50–3,489.85)	−0.56 (−0.64 to −0.48)
Australasia	3,994.95 (3,887.71–4,085.43)	2,928.61 (2,780.51–3,070.41)	−1.21 (−1.27 to −1.14)
Caribbean	3,473.97 (3,179.79–3,665.43)	3,385.00 (2,906.72–3,930.62)	0.01 (−0.06 to 0.09)
Central Asia	4,067.90 (3,971.65–4,167.04)	3,243.26 (2,954.42–3,571.76)	−0.84 (−0.92 to −0.75)
Central Europe	4,655.22 (4,570.06–4,751.17)	4,066.16 (3,577.18–4,611.01)	−0.49 (−0.56 to −0.41)
Central Latin America	2,901.28 (2,828.22–2,987.83)	2,516.85 (2,163.53–2,913.81)	−0.61 (−0.67 to −0.56)
Central Sub-Saharan Africa	3,146.21 (2,621.71–3,776.61)	2,752.50 (2,155.91–3,393.16)	−0.54 (−0.60 to −0.48)
East Asia	4,666.71 (4,137.56–5,198.17)	3,425.93 (2,961.42–3,936.25)	−1.22 (−1.33 to −1.12)
Eastern Europe	4,449.39 (4,374.91–4,519.76)	3,542.74 (3,218.95–3,909.39)	−1.22 (−1.42 to −1.02)
Eastern Sub-Saharan Africa	3,206.46 (2,822.65–3,600.78)	2,896.14 (2,480.25–3,357.86)	−0.40 (−0.45 to −0.36)
High-income Asia Pacific	3,648.13 (3,545.09–3,714.84)	2,534.63 (2,372.35–2,644.68)	−1.40 (−1.48 to −1.32)
High-income North America	4,225.14 (4,097.32–4,317.90)	3,214.82 (3,094.05–3,328.72)	−1.13 (−1.19 to −1.07)
North Africa and Middle East	2,740.79 (2,451.05–2,981.52)	2,513.98 (2,252.71–2,809.14)	−0.20 (−0.33 to −0.08)
Oceania	3,083.09 (2,633.81–3,609.51)	3,301.90 (2,672.17–4,128.04)	0.27 (0.25 to 0.30)
South Asia	2,420.92 (2,196.62–2,642.26)	2,398.76 (2,147.83–2,671.79)	−0.14 (−0.21 to −0.06)
Southeast Asia	3,034.82 (2,724.24–3,285.57)	2,904.32 (2,578.39–3,270.34)	−0.26 (−0.31 to −0.22)
Southern Latin America	4,465.01 (4,368.83–4,562.33)	3,600.71 (3,455.70–3,755.16)	−0.83 (−0.88 to −0.79)
Southern Sub-Saharan Africa	3,416.95 (3,083.00–3,771.00)	3,319.95 (3,039.17–3,584.79)	−0.15 (−0.42 to 0.12)
Tropical Latin America	3,409.72 (3,294.24–3,584.11)	2,877.51 (2,739.80–2,997.93)	−0.57 (−0.65 to −0.49)
Western Europe	4,146.16 (4,045.51–4,218.42)	3,144.12 (3,005.05–3,250.40)	−1.06 (−1.09 to −1.02)
Western Sub-Saharan Africa	2,333.68 (2035.85–2,668.73)	2,541.79 (2,103.60–2,982.79)	0.39 (0.35 to 0.43)

The estimated association between SDI and ASRs due to neoplasms, shown as the blue line in Figure 2, was a gradual increase for ASRs as SDI increased. Meanwhile, a positive relationship was observed between SDI and YLD-YLL ratio (Figure 3). As SDI increased, YLD accounted for a progressively larger proportion of the neoplasms burden. In addition, a negative relationship was detected between EAPC and SDI (Supplementary Figure S4), which meant countries with higher SDI have experienced a more rapid decrease in ASRs of neoplasms from 1990 to 2019. The ASMR and ASDR of neoplasms showed declining trends in each gender (Table 2; Supplementary Figure S5). However, SDI was positively correlated with gender differences in burden of neoplasms, suggesting that the difference in ASRs between men and women increases with socio-demographic development (Supplementary Figure S6).

### Disease Burden Due to Chronic Respiratory Diseases

Since 1990 the global ASMR of CRD has decreased, with an EAPC of −2.10 (95% CI of −2.19 to −2.00). The positive EAPC is found in Nicaragua (EAPC = 1.26; 95% CI 0.99 to 1.62), followed by Greece. The most pronounced decline was in Singapore (−5.99; −6.31 to −5.66), followed by Belarus. Also, the ASDR is on a downward trend globally, with an EAPC of −1.87 (−1.93 to −1.81). Turkmenistan showed the significant decrease in ASDR (EAPC = −4.50, −4.90 to −4.11), followed by Singapore. On the other hand, Georgia had the highest EAPC of 0.81 (0.44 to 1.18) (Supplementary Figure S2).

Over the study period, decreasing trends were observed in both ASMRs and ASDRs across five SDI regions. The most rapid decreases in ASRs were detected in high-middle and middle SDI regions (Table 3). In addition, the decline in high SDI regions has stagnated in recent years. However, during the study period, ASRs in high SDI regions remained the lowest (Supplementary Figure S3). For 21 GBD regions, except for High-income North America, all the regions showed decreasing trends in ASMRs and ASDRs. The lowest EAPC in ASMR was observed in East Asia (−4.45; −4.65 to −4.26) and Eastern Europe (−4.03; −4.37 to −3.69). The lowest EAPC in ASDR was also observed in East Asia (−4.16; −4.31 to −4.00) and Eastern Europe (−3.36; −3.65 to −3.07) (Table 3). The estimated relationship between ASRs of CRD and SDI, shown as the blue line in Figure 2, was a gradual decrease for ASRs as SDI increased. A positive relationship was observed between SDI and YLD-YLL ratio, with more rapid growth at the higher SDI levels (Figure 3). Besides, despite the decreasing trends in ASR for both males and females, males have a higher mortality rate than females. With the increase of SDI, the CRD showed the similar trends of gender differences as CVD to some extent (Supplementary Figure S6).

**TABLE 3:** The age standardized mortality rate (A) and age standardized Disability adjusted life years rate (B) and corresponding temporal trends of chronic respiratory diseases. Global Burden of Diseases Study, 21 Global Burden of Diseases regions, 1990–2019.

A	Age standardized mortality rates (95% UI)		EAPC (95% CI) 1990–2019
	1990	2019	
Global	87.89 (73.87–95.10)	51.28 (45.90–55.51)	−2.10 (−2.19 to −2.00)
Sex			
Male	116.75 (102.61–126.76)	66.72 (60.55–73.06)	−2.14 (−2.24 to −2.04)
Female	67.84 (51.59–75.41)	39.73 (33.24–44.75)	−2.11 (−2.21 to −2.02)
SDI			
Low	114.69 (96.34–133.08)	87.82 (74.17–97.67)	−0.98 (−1.08 to −0.88)
Low-middle	160.43 (135.86–179.49)	107.29 (90.10–120.82)	−1.47 (−1.55 to −1.40)
Middle	134.26 (106.75–147.00)	59.82 (52.30–66.61)	−3.06 (−3.20 to −2.92)
High-middle	80.81 (64.52–87.92)	33.22 (29.45–39.31)	−3.53 (−3.70 to −3.35)
High	30.30 (28.30–33.21)	24.64 (21.49–26.07)	−0.87 (−0.94 to −0.80)
GBD region			
Andean Latin America	33.72 (30.10–40.56)	26.84 (20.79–32.10)	−0.27 (−0.45 to −0.09)
Australasia	36.52 (33.93–38.72)	24.82 (20.92–27.50)	−1.57 (−1.76 to −1.38)
Caribbean	28.30 (24.92–31.68)	26.87 (22.23–31.67)	−0.18 (−0.25 to −0.11)
Central Asia	51.12 (43.53–54.07)	39.41 (35.47–46.52)	−1.48 (−1.77 to −1.18)
Central Europe	36.96 (33.94–38.21)	19.12 (16.63–22.05)	−2.23 (−2.39 to −2.08)
Central Latin America	42.34 (37.85–44.43)	33.69 (28.27–38.66)	−1.00 (−1.09 to −0.92)
Central Sub-Saharan Africa	86.5 (60.43–123.44)	65.80 (44.51–104.69)	−0.94 (−1.09 to −0.80)
East Asia	221.48 (166.31–246.45)	67.48 (57.8–82.27)	−4.45 (−4.65 to −4.26)
Eastern Europe	39.44 (30.85–42.36)	16.23 (14.22–20.72)	−4.03 (−4.37 to −3.69)
Eastern Sub-Saharan Africa	63.20 (53.59–75.14)	42.40 (36.86–48.27)	−1.49 (−1.53 to −1.44)
High-income Asia Pacific	24.00 (20.85–25.25)	12.31 (10.40–13.74)	−2.59 (−2.74 to −2.45)
High-income North America	31.29 (29.23–36.15)	36.24 (29.59–38.58)	0.38 (0.21 to 0.55)
North Africa and Middle East	53.80 (47.11–61.58)	36.10 (30.90–40.31)	−1.24 (−1.32 to −1.17)
Oceania	201.85 (164.72–235.52)	166.28 (133.34–202.63)	−0.69 (−0.73 to −0.65)
South Asia	179.62 (154.45–204.39)	118.75 (97.56–135.84)	−1.54 (−1.69 to −1.40)
Southeast Asia	90.37 (70.75–102.45)	53.72 (46.49–59.45)	−1.84 (−1.92 to −1.75)
Southern Latin America	32.72 (30.22–37.11)	32.56 (28.24–35.95)	−0.13 (−0.34 to 0.09)
Southern Sub-Saharan Africa	65.65 (55.94–77.28)	49.21 (44.37–54.24)	−1.20 (−1.65 to −0.75)
Tropical Latin America	54.94 (49.56–58.05)	34.10 (30.50–38.25)	−2.01 (−2.18 to −1.85)
Western Europe	31.00 (28.87–33.56)	22.45 (19.62–24.03)	−1.21 (−1.31 to −1.12)
Western Sub-Saharan Africa	54.64 (45.14–63.75)	39.14 (33.48–44.62)	−0.96 (−1.03 to −0.89)

B	Age Standardized DALY Rates (95% UI)		EAPC (95% CI) 1990–2019
	1990	2019	
Global	2,107.59 (1836.22–2,266.19)	1,293.74 (1,182.99–1,403.57)	−1.87 (−1.93 to −1.81)
Sex			
Male	2,601.44 (2,288.63–2,817.61)	1,538.69 (1,399.47–1,690.3)	−1.99 (−2.05 to −1.92)
Female	1,726.15 (1,395.38–1896.69)	1,093.03 (965.72–1,208.96)	−1.78 (−1.85 to −1.72)
SDI			
Low	2,749.84 (2,370.45–3,068.74)	2048.37 (1802.01–2,247.14)	−1.06 (−1.14 to −0.98)
Low-middle	3,504.76 (2,997.35–3,836.69)	2,314.24 (2029.67–2,562.08)	−1.50 (−1.55 to −1.45)
Middle	2,712.23 (2,230.86–2,951.54)	1,316.93 (1,199.96–1,449.4)	−2.73 (−2.81 to −2.64)
High-middle	1,767.21 (1,507.45–1926.22)	837.00 (750.92–948.78)	−3.01 (−3.16 to −2.87)
High	1,109.21 (961.09–1,283.53)	924.07 (797.2–1,067.03)	−0.66 (−0.72 to −0.60)
GBD region			
Andean Latin America	1,035.12 (903.56–1,176.30)	680.12 (559.51–811.82)	−1.24 (−1.42 to −1.06)
Australasia	1,376.88 (1,179.57–1,606.72)	946.08 (793.37–1,128.11)	−1.63 (−1.77 to −1.49)
Caribbean	1,082.25 (917.66–1,261.62)	960.2 (797.86–1,118.46)	−0.39 (−0.44 to −0.35)
Central Asia	1,290.81 (1,147.5–1,380.62)	937.22 (847.05–1,056.72)	−1.74 (−2.00 to −1.47)
Central Europe	1,072.36 (963.33–1,197.13)	678.01 (583.53–787.39)	−1.64 (−1.77 to −1.52)
Central Latin America	1,030.45 (936.74–1,132.59)	784.43 (686.77–890.61)	−1.15 (−1.23 to −1.07)
Central Sub-Saharan Africa	2,152.87 (1,630.09–2,792.55)	1,625.00 (1,229.32–2,246.80)	−0.99 (−1.11 to −0.88)
East Asia	3,845.41 (2,938.76–4,279.44)	1,270.89 (1,120.48–1,470.61)	−4.16 (−4.31 to −4.00)
Eastern Europe	1,111.67 (943.64–1,234.04)	537.15 (467.11–633.55)	−3.36 (−3.64 to −3.07)
Eastern Sub-Saharan Africa	1822.22 (1,588.45–2047.46)	1,231.52 (1,082.84–1,392.41)	−1.46 (−1.50 to −1.43)
High-income Asia Pacific	837.64 (713.06–984.42)	468.37 (392.53–561.90)	−2.34 (−2.50 to −2.19)
High-income North America	1,309.32 (1,138.88–1,514.62)	1,374.00 (1,180.95–1,570.24)	0.29 (0.25 to 0.34)
North Africa and Middle East	1,403.74 (1,252.64–1,560.45)	1,033.42 (906.68–1,149.27)	−1.03 (−1.07 to −0.99)
Oceania	4,495.51 (3,837.12–5,182.71)	3,677.62 (3,020.87–4,477.19)	−0.69 (−0.72 to −0.67)
South Asia	3,830.22 (3,350.24–4,261.91)	2,559.27 (2,206.93–2,879.03)	−1.47 (−1.55 to −1.39)
Southeast Asia	2,163.45 (1,822.55–2,373.96)	1,383.12 (1,235.32–1,511.65)	−1.59 (−1.64 to −1.55)
Southern Latin America	1,006.41 (889.66–1,149.56)	942.10 (817.88–1,086.82)	−0.36 (−0.46 to −0.25)
Southern Sub-Saharan Africa	1,861.61 (1,647.57–2094.22)	1,387.64 (1,263.76–1,520.81)	−1.20 (−1.55 to −0.85)
Tropical Latin America	1,366.72 (1,238.72–1,516.07)	909.91 (808.60–1,037.21)	−1.81 (−1.97 to −1.66)
Western Europe	1,034.21 (899.30–1,205.75)	769.63 (660.74–900.99)	−1.10 (−1.18 to −1.02)
Western Sub-Saharan Africa	1,524.38 (1,310.15–1739.37)	1,128.76 (986.27–1,272.71)	−0.95 (−1.00 to −0.90)

## Discussion

This study mainly analyzed the spatial and temporal patterns of the burden caused by the three major NCDs (cardiovascular diseases, neoplasms and chronic respiratory diseases), and examined the association between ASMRs, ASDRs and SDI. During the study period, the transition in disease burden from CMNN to NCDs was pronounced, with the proportion of NCDs deaths increasing from 56.75% to 74.36%. After removing the effects of population growth and aging, the ASRs showed significant declining trends globally, indicating that the prevention and control of NCDs was effective to a certain extent. However, the gaps between regions and countries were still obvious.

The transition in burden patterns would pose significant challenges to many lower SDI countries [23]. Many of these countries have been focusing on CMNN rather than the prevention or long-term care needed for NCDs [24, 25]. Evidence has indicated that low- and middle-income countries invested a large proportion of government health funds in CMNN rather than NCDs [26]. Additionally, government expenditure on health was relatively lower compared to higher-income countries [27, 28]. Before the economic and social development reaches a higher level, the coverage of some NCDs interventions may lag behind that of CMNN interventions in lower-income countries [29]. The SDI was identified as a critical factor affecting the mortality and DALY rate, which may explain regional variations to a certain extend [30]. In addition, social conditions, behavioral and metabolic risk factors (e.g., education, household income, unemployment, smoking, obesity, dietary risk, air pollution and hypertension) could also contribute to inequalities in disease burden among different SDI countries and regions [31]. According to the most recent United Nations’ report, most low- and middle-income countries are falling short of meeting SDG 3.4 for NCD mortality rate. Despite wide variations in global NCD epidemiology and health system capacity, progress in the prevention and targeting of risk factors for NCDs (e.g., tobacco, alcohol consumption, and hypertension) in some countries suggests that cross-sectoral policies on tobacco, alcohol, and unhealthy diets are relevant and necessary in all countries. In response to these risk factors, the United Nations released the “Disease Control Priorities,” which give a model list of cost-effective treatments that are realistic to execute in low- and middle-income countries and may have the potential to address a major disease burden [32].

For CVD, the ASRs of CVD in the high SDI region remained the lowest worldwide, which may be related to better access to health services, early screening, and lifesaving medications in the high SDI region [33, 34]. Whereas the ASRs of CVD in the high-middle SDI region did not match the expected value-based SDI. Our study showed that CVD in the high-middle SDI region, once the highest in the world, was still above the global level, although it has shown an obvious downward trend during the study period. Most high-middle SDI countries are developing countries, like India and China, which have experienced rapid economic and population growth in a short time. And the aging of the population is becoming more serious. Meanwhile, people in developing countries are increasingly exposed to risk factors due to globalization and urbanization [35]. Compared to GBD 1990, behavioral and metabolic factors have become the leading factors in CVD burden in the high-middle SDI region. While interventions to address these factors cannot achieve improvement in a short time [36]. Meanwhile, the absence of universal healthcare coverage, lack of access to public health education, prevention programs and primary care may be other contributors to the relatively higher burden of CVD in high-middle SDI regions [31, 35]. While these conditions would be even worse in low, low-middle and middle SDI countries.

Over time, the downward trends of ASRs due to neoplasms across all SDI regions were encouraging. However, unlike CVD and CRD, the burden of neoplasms increased to a certain extent as SDI rose. In the low SDI region, population growth was major contributor to the burden of neoplasms. In contrast, the burden of neoplasms may be mainly driven by population aging in high-middle and high SDI regions [37, 38]. Socio-economic disparities in neoplasms burden varied by neoplasms type, especially those preventable neoplasms [39, 40]. Cervical neoplasms are likely the best example, and the burden varies considerably across SDI [37]. As for preventable neoplasms, early screening and vaccination can lead to good outcomes, while socio-economic deprivations were associated with lower neoplasms screening prevalence, later-stage diagnosis, and high-risk factors [41, 42]. Additionally, previous studies showed that in many lower SDI countries, the burden of neoplasms related to infectious aetiologies and poor nutrition was declining, whereas neoplasms associated with obesity and alcohol consumption were becoming more common [8, 43]. Meanwhile, the neoplasms burden caused by unsafe sex was still severe in low and low-middle SDI regions, especially for females. This requires policymakers to formulate more targeted policies.

For chronic respiratory diseases, negative relationships between SDI and ASRs were observed. High SDI region showed the lowest burden of CRD, and the high-middle SDI region showed the most pronounced downward trend. Differences in SDI among regions caused healthcare disparities, which included poor healthcare access, inability to timely diagnose advanced lung disease, inadequate availability of advanced diagnostic modalities, and delay in referral to advanced lung disease centers. All of these ultimately led to treatment and clinical differences [44]. In addition, as a major risk factor of CRD, the smoking prevalence has decreased worldwide since 1990, but the progress in different regions was not universal [12, 14, 45]. Meanwhile, due to the lag effect and secondhand smoke, the burden of CRD contributed to smoking was still higher in low, low-middle, and middle SDI regions [46]. Additionally, exposure to air pollution has increased since 1990, especially in lower SDI regions [47].

Furthermore, to investigate the changes in YLDs proportion, the relationships between SDI and YLD-YLL ratio were examined. Similar positive relationships were observed for three diseases. We were expected to see that with increasing SDI, the proportion of YLDs in DALYs was increasing, which was consistent with the previous study [13]. Higher SDI means more access to and use of health services, broad-coverage universal health insurance, earlier screening, and more convenience to primary care, contributing to the higher proportion of YLDs compared to lower SDI regions [31, 44]. Relationships between sex and NCDs were complex. This study showed that between 1990 and 2019, the number of deaths and DALYs of three diseases for each sex tended to increase, but the age standardized mortality and age standardized DALY rates for each sex were on the decreasing trends. For CVD and CRD, the gender difference in ASRs tended to increase and then decrease with SDI, implying that the gender difference is likely to be most pronounced in countries with middle SDI. For neoplasms, gender differences of ASRs were increasing as SDI increased. What needs to pay attention to is that in some lower SDI countries, the burden of CVD and neoplasms is higher in females [48]. Females in these countries lack access to education, health-related information, and health services. Meanwhile, they often serve as caregivers for family members with NCDs and have limited autonomy and control over their health decisions, exacerbating the disease burden in females [49, 50].

GBD studies come up with comprehensive quality global disease burden estimates, yet several limitations should be noted. As a secondary study, we paid attention to the spatial and temporal patterns of disastrous burden of CVD, neoplasms and CRD and examined the association with SDI. The accuracy and robustness of GBD dataset largely depend on the quality and quantity of data used in the modelling. Civil registration and statistics systems are key sources of vital statistics for mortality rates, but the population coverage with these systems has been disappointing in some low-income regions, which may lead to underestimating the burden of disease, although GBD has conducted many adjusted methods to reduce such bias. Besides, this study mainly described the overall trends of three main NCDs, while variations in the mortality and DALY rates may partially represent the detection biases linked with the screening and diagnostic protocol’s modifications rather than factual changes in age-specific rates. Meanwhile, EAPCs were used to evaluate the changing trend for disease burden of cardiovascular diseases, neoplasms and chronic respiratory diseases over the overall period from 1990 to 2019, which might ignore the details of burden changes in some areas. Additionally, ecological fallacy might emerge since our study is based on the population level.

### Conclusion

NCDs have been the main contributor to the burden of disease. CVD, neoplasms and CRD remained the major public health concern worldwide. Despite the impact of population growth and ageing, the burden of the major NCDs has improved globally. However, regional and gender disparities were observed in our study, as well as stagnation in the decline of disease burden in the high SDI region. Meanwhile, low SDI is a major impediment to progress in burden reduction in developing countries. In addition, ageing and risk exposures, including tobacco, air pollution and metabolic factors, may be the major drivers and should receive more attention and supportive policies to reduce premature deaths, mainly in low and middle SDI countries with inadequate healthcare provision.

## Abbreviations

ASDRs, age standardized DALY rates; ASMRs, age standardized mortality rates; ASRs, age standardized rates; CMNN, communicable, maternal, neonatal, and nutritional causes; CRD, chronic respiratory diseases; CVD, cardiovascular diseases; DALY, disability adjusted life years; EAPCs, estimated annual percentage changes; GBD, Global Burden of Diseases; NCDs, non-communicable diseases; QR, quantile regression; RCS, limited cubic splines; SDI, socio-demographic Index; YLDs, years lived with disability; YLLs, years of life lost.
