# Supplementary material for: Global Epidemiological Patterns in the Burden of Main Non-Communicable Diseases, 1990–2019: Relationships With Socio-Demographic Index
Source: Int J Public Health. 2023 Jan 16;68:1605502. doi: 10.3389/ijph.2023.1605502 (PMC9884670; doi:10.3389/ijph.2023.1605502)
Supplement: Supplementary file 1 [file DataSheet1.docx]

Supplementary Material

## Supplementary Figures


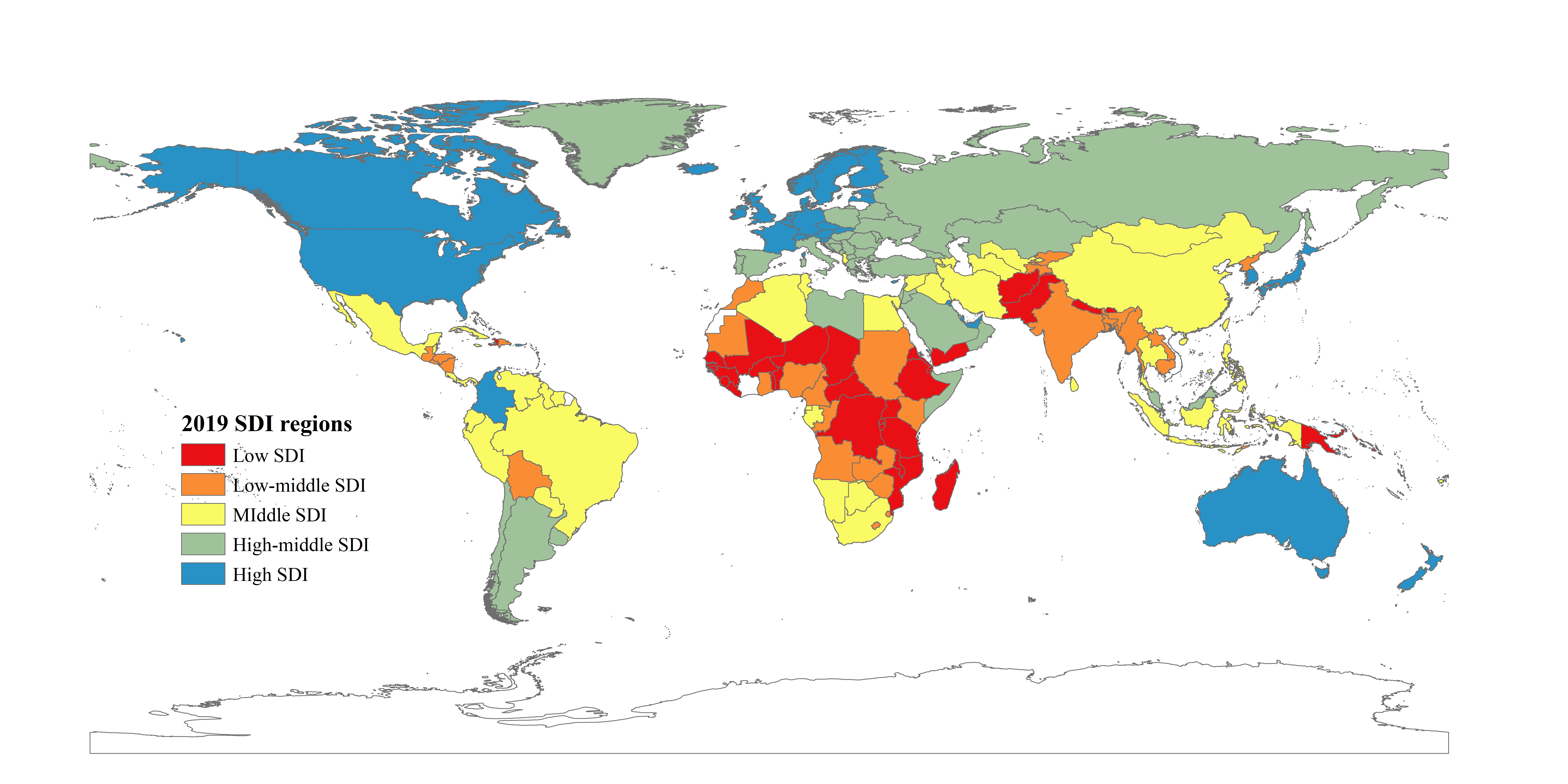


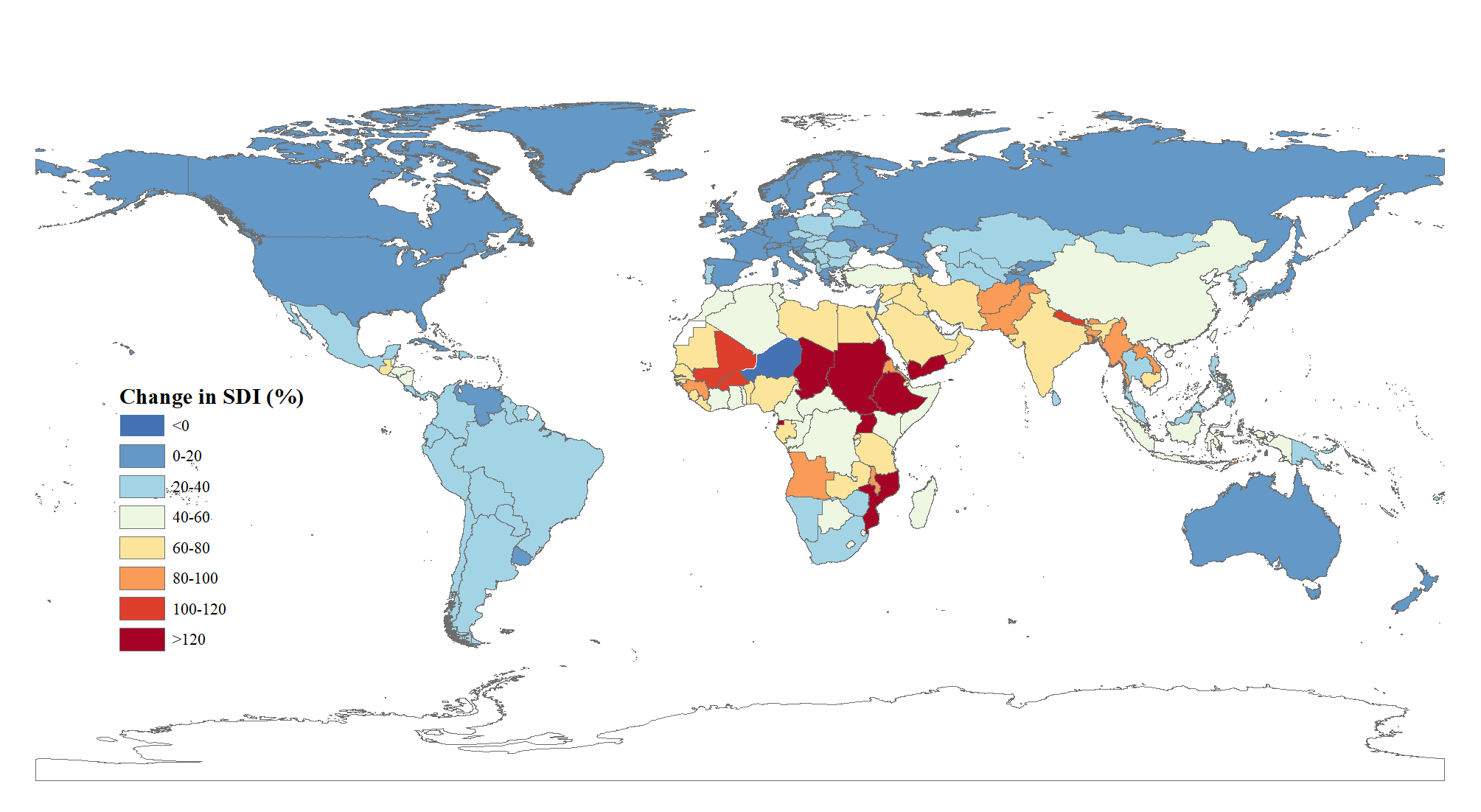


**Supplementary Figure 1.** Geographical distribution of socio-demographic index in 2019 and change in socio-demographic index (%) by country or territory from 1990 to 2019.

**

**

**Supplementary Figure 2.** EAPC in ASMR (left) and ASDR (right) of cardiovascular diseases, neoplasms and chronic respiratory diseases in 204 countries and territories from 1990 to 2019.


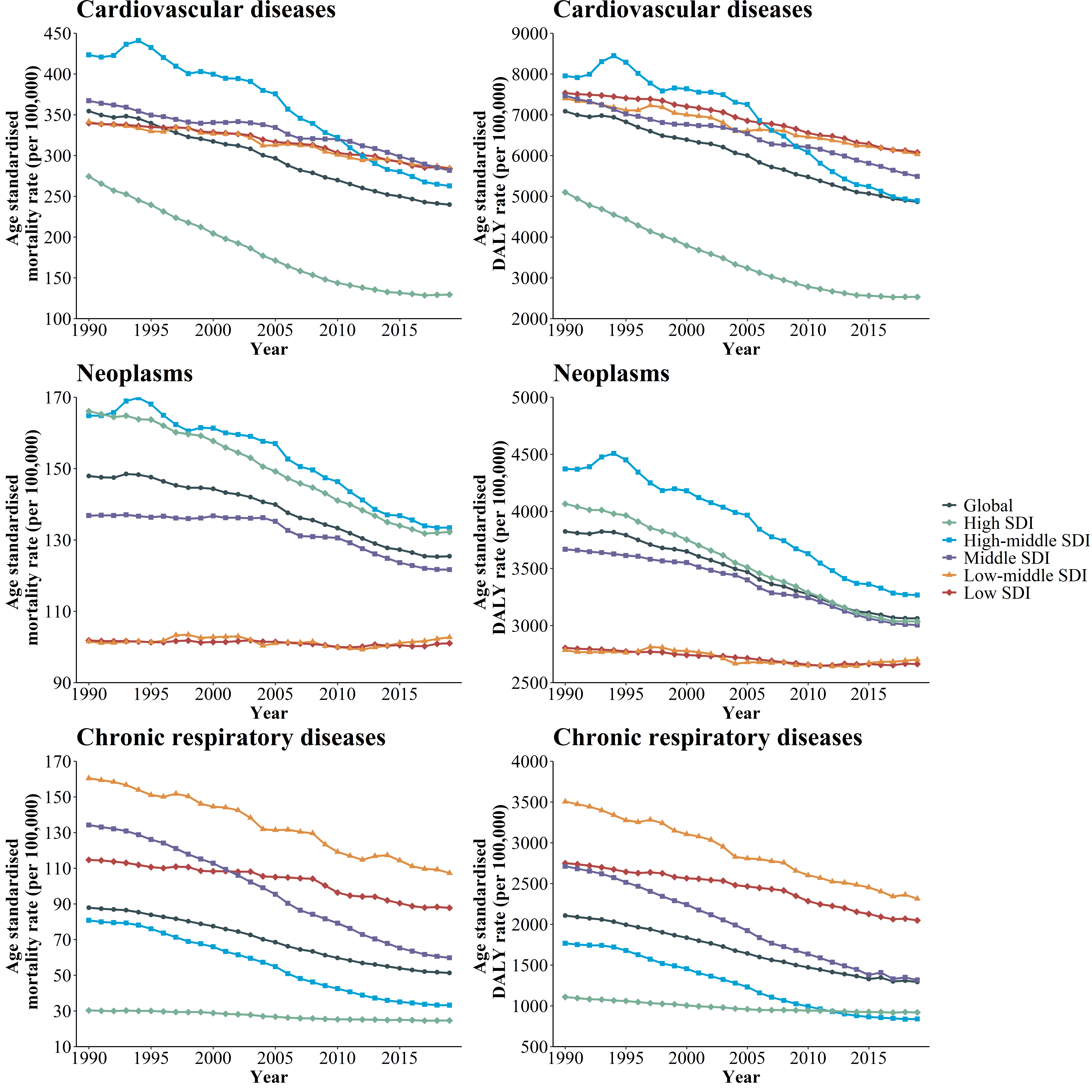


**Supplementary figure 3.** Age standardized mortality rates (left) and age standardized DALY rates (right) per 100 000 people of cardiovascular diseases, neoplasms and chronic respiratory diseases by SDI regions from 1990 to 2019


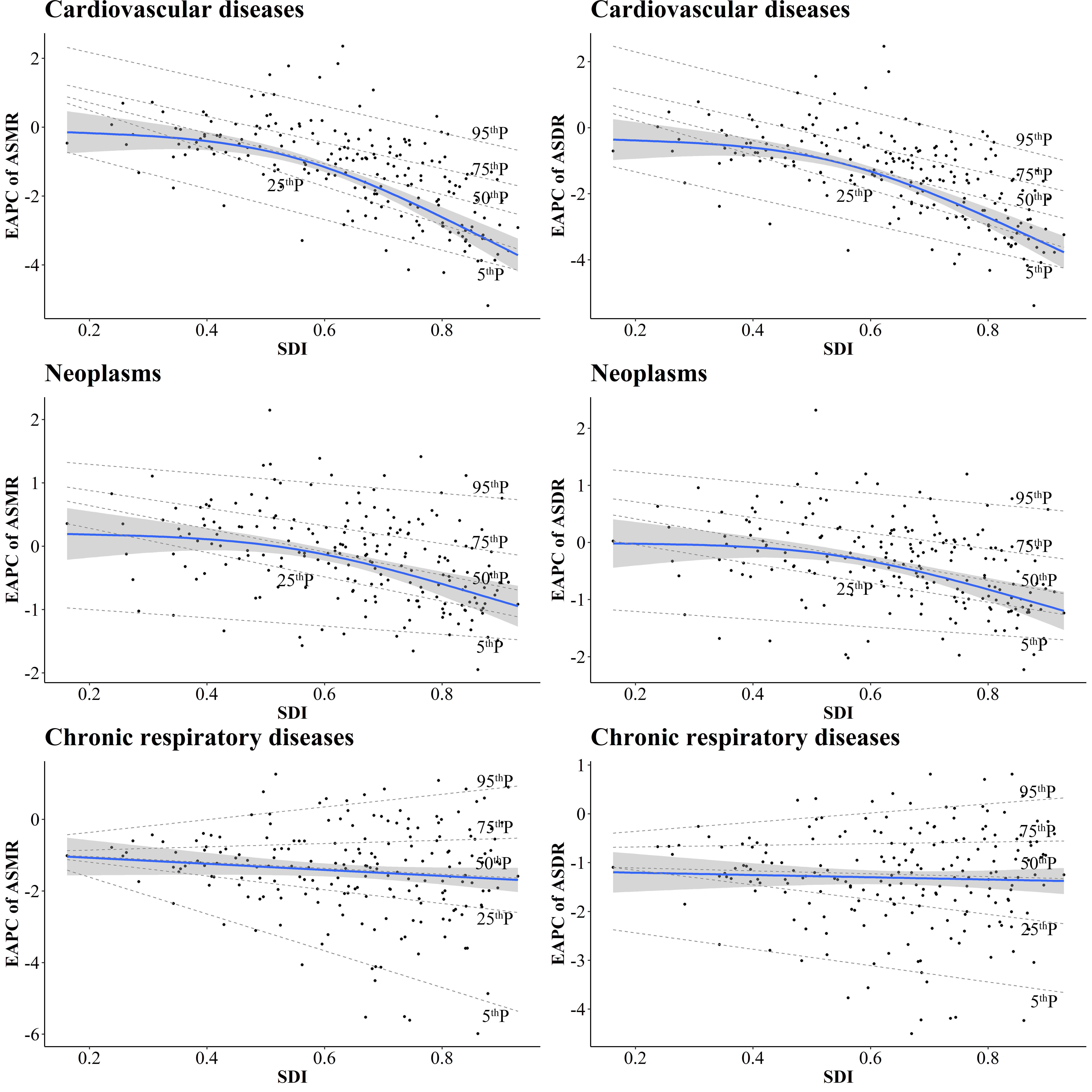


**Supplementary figure 4.** The correlation between the SDI and EAPC of ASMR (left) and ASDR (right) in cardiovascular diseases, neoplasms and chronic respiratory diseases.


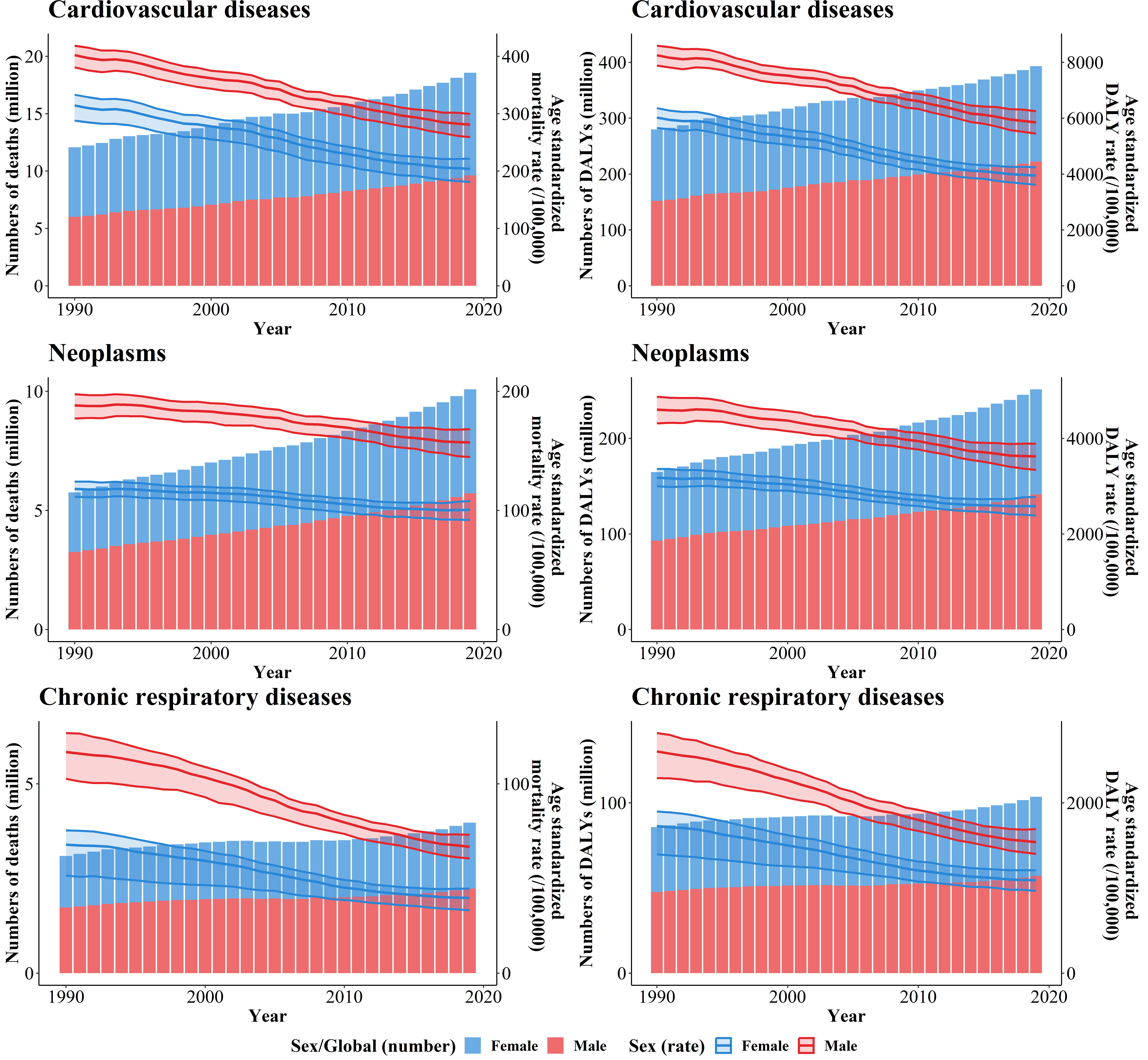


**Supplementary figure 5.** Deaths, DALYs, age standardized mortality rates and age standardized DALY rates per 100 000 people of cardiovascular diseases, neoplasms and chronic respiratory diseases globally from 1990 to 2019


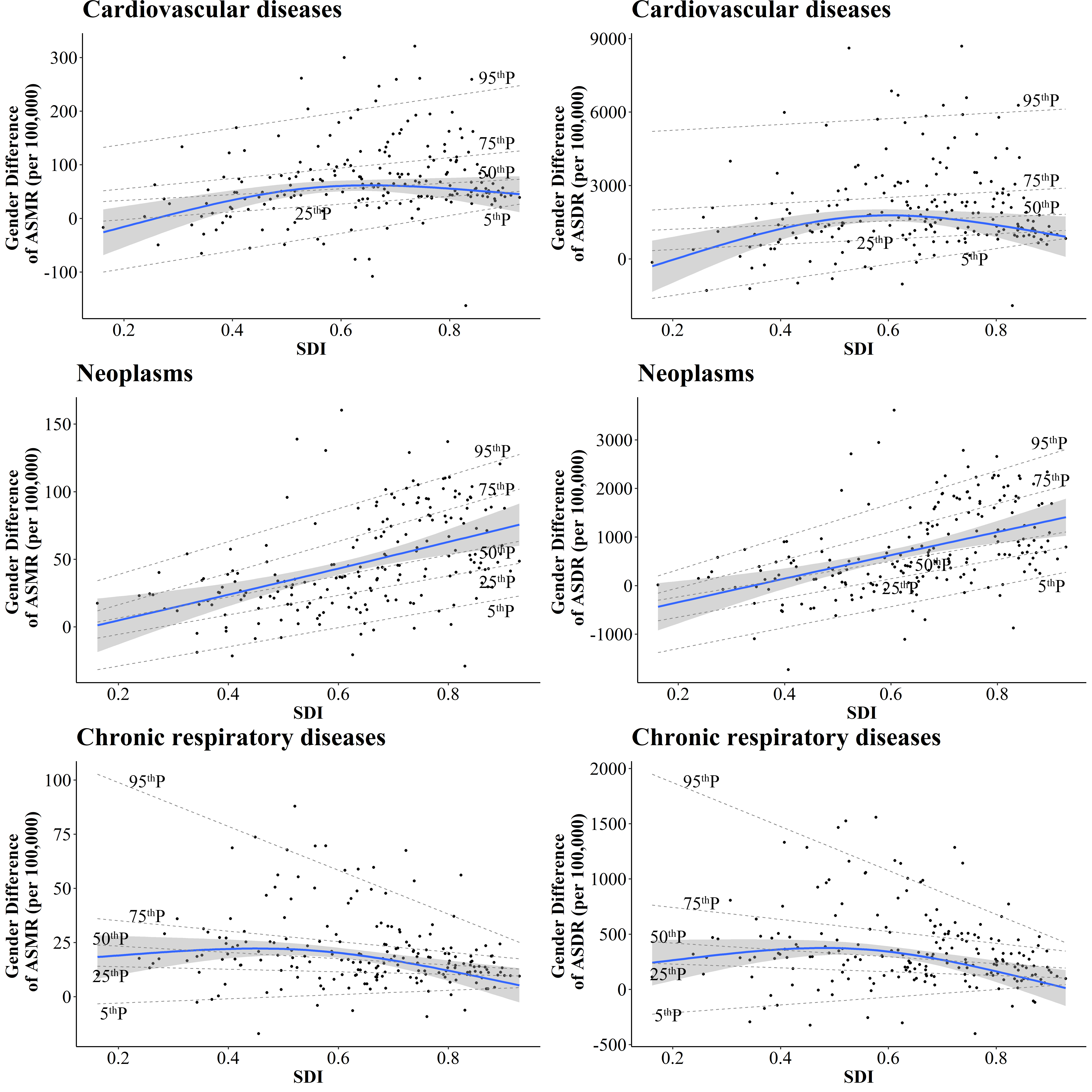


**Supplementary figure 6.** The correlation between the SDI and gender difference of ASMR (left) and ASDR (right) in cardiovascular diseases, neoplasms and chronic respiratory diseases in 2019

# Supplementary Tables

**Supplementary Table 1.** The ICD-10 code for cardiovascular diseases, neoplasms and chronic respiratory diseases.

| Disease | ICD10 |
| --- | --- |
| Cardiovascular diseases | B33.2-B33.24, D86.85, G45-G46.8, I01-I01.9, I02.0, I05-I09.9, I11-I11.2, I11.9, I20-I21.6, I21.9-I27.0, I27.2-I28.9, I30-I38.0, I39-I41.8, I42-I43.8, I44-I44.8, I45-I52.8, I60-I64, I64.1, I65-I83.93, I86-I89.0, I89.9, I95.0-I95.1, I98, I98.8-I99.9, K75.1, R00-R01.2, Z01.3-Z01.31, Z03.4-Z03.5, Z13.6, Z52.7, Z82.3-Z82.49, Z86.7-Z86.79, Z94.1-Z94.3, Z95-Z95.9 |
| Neoplasms | C00-C07, C08-C19.0, C20, C21-C21.8, C22-C22.4, C22.7-C23, C24-C26.1, C26.8-C26.9, C30-C30.1, C31-C33, C34-C34.92, C37-C37.0, C38-C39.9, C40-C41.4, C41.8-C41.9, C43-C45.2, C45.7, C45.9, C47-C4A, C50-C50.629, C50.8-C52, C53-C54.3, C54.8-C56.2, C56.9-C58.0, C60-C64.2, C64.9-C69.92, C70-C70.1, C70.9-C73, C74-C75.5, C75.8-C79.9, C80-C81.49, C81.7-C81.79, C81.9-C85.29, C85.7-C86.6, C88-C90.32, C91-C93.7, C93.9-C95.2, C95.7-C97.9, D00-D24.9, D26.0-D39.9, D4-D49.9, E34.0, K51.4-K51.419, K62.0-K62.3, K63.5, N60-N60.99, N84.0-N84.1, N87-N87.9, Z03.1, Z08-Z09.9, Z12-Z12.9, Z80-Z80.9, Z85-Z85.9, Z86.0-Z86.03 |
| Chronic respiratory diseases | D86-D86.2, D86.9, G47.3-G47.39, J30-J35.9, J37-J39.9, J41-J42.4, J43-J46.0, J47-J47.9, J60-J68.9, J70.8-J70.9, J80-J80.9, J82, J84-J84.9, J90-J90.0, J91, J91.8-J93.12, J93.8-J94.9, J96-J96.92, J98-J99.8, R05.0-R06.9, R09-R09.89, R84-R84.9, R91-R91.8, Z82.5 |

**Supplementary Table 2.** Quantile regression estimates predicting change in cardiovascular diseases, neoplasms and chronic respiratory diseases due to SDI on conditional distributions across quantiles for the worldwide regions

| Cardiovascular diseases |  | ASMR | ASDR | YLD-YLL ratios |
| --- | --- | --- | --- | --- |
|  |  | Coefficient (95%CI) | Coefficient (95%CI) | Coefficient (95%CI) |
| 5th percentile | Intercept | 321.75* (264.13, 342.66) | 6609.67* (5333.91, 7297.04) | 0.00 (-0.03, 0.04) |
|  | SDI | -280.27* (-307.13, -218.55) | -5973.02* (-7042.62, -4273.39) | 0.07* (0.00, 0.11) |
| 25th percentile | Intercept | 383.44* (356.15, 397.46) | 8089.28* (7578.48, 8411.59) | 0.02 (-0.01, 0.04) |
|  | SDI | -297.14* (-313.12, -256.22) | -6800.22* (-7229.66, -6176.29) | 0.08* (0.03, 0.10) |
| 50th percentile | Intercept | 406.13* (338.21, 523.15) | 9223.67* (7499.62, 11555.34) | 0.02* (0.00, 0.04) |
|  | SDI | -215.73* (-387.99, -108.21) | -6285.39* (-9871.45, -3311.99) | 0.10* (0.06, 0.14) |
| 75th percentile | Intercept | 452.31* (385.11, 742.94) | 10812.09* (8284.05, 19356.68) | 0.03* (0.02, 0.04) |
|  | SDI | -149.35* (-482.9, -37.19) | -5875.41* (-11622.77, -2637.78) | 0.12* (0.11, 0.16) |
| 95th percentile | Intercept | 856.67* (534.06, 1331.17) | 22084.81* (13998.86, 32684) | 0.01* (0.00, 0.02) |
|  | SDI | -473.01 (-1006.24, 85.16) | -16904.24* (-31190.26, -3368.65) | 0.23* (0.21, 0.24) |
| Neoplasms |  | ASMR | ASDR | YLD-YLL ratios |
|  |  | Coefficient (95%CI) | Coefficient (95%CI) | Coefficient (95%CI) |
| 5th percentile | Intercept | 82.23* (39.93, 93.21) | 2208.39* (734.45, 2722.13) | 0.00 (-0.02, 0.01) |
|  | SDI | 9.67 (-31.09, 39.67) | -124.2 (-1272.78, 1704.23) | 0.02* (0.02, 0.05) |
| 25th percentile | Intercept | 92.59* (78.76, 98.01) | 2457.61* (2140.79, 2619.57) | -0.01 (-0.02, 0.00) |
|  | SDI | 26.84* (17.04, 53.41) | 423.14* (13.52, 823.43) | 0.05* (0.04, 0.06) |
| 50th percentile | Intercept | 92.41* (82.04, 97.99) | 2513.52* (2360.97, 2905.13) | -0.01* (-0.01, -0.01) |
|  | SDI | 52.41* (43.68, 67.69) | 863.82* (187.51, 1127.07) | 0.06* (0.05, 0.07) |
| 75th percentile | Intercept | 105.73* (89.66, 136.74) | 3145.22* (2541.08, 3908.82) | -0.01* (-0.02, -0.01) |
|  | SDI | 60.12* (1.67, 87.16) | 759.61 (-286.05, 1761.16) | 0.08* (0.08, 0.08) |
| 95th percentile | Intercept | 168.82* (115.38, 199.23) | 4416.12* (3497.86, 6899.2) | -0.01* (-0.01, -0.01) |
|  | SDI | 21.62 (-20.78, 123.43) | 246.36* (-3281.99, 1801.56) | 0.09* (0.08, 0.09) |
| Chronic respiratory  diseases |  | ASMR | ASDR | YLD-YLL ratios |
|  |  | Coefficient (95%CI) | Coefficient (95%CI) | Coefficient (95%CI) |
| 5th percentile | Intercept | 49.2* (40.81, 55.93) | 1292.02* (908.99, 1466.57) | -0.20 (-0.70, 0.01) |
|  | SDI | -46.62* (-59.37, -35.16) | -1064.02* (-1320.51, -800.34) | 0.66* (0.29, 1.39) |
| 25th percentile | Intercept | 63.1* (54.47, 67.19) | 1639.83* (1524.9, 1783.88) | -0.41* (-0.6, -0.02) |
|  | SDI | -58.67* (-64.6, -46.52) | -1323.8* (-1534.45, -1152.54) | 1.37* (0.72, 1.80) |
| 50th percentile | Intercept | 75.12* (65.81, 82.38) | 1997.19* (1764.13, 2120.47) | -0.28* (-0.53, -0.17) |
|  | SDI | -64.78* (-74.81, -54.8) | -1610.76* (-1777.11, -1138.82) | 1.48* (1.29, 1.82) |
| 75th percentile | Intercept | 108* (92.01, 144.92) | 2420.07* (2241.58, 3165.78) | -0.22* (-0.38, -0.13) |
|  | SDI | -94.34* (-133.01, -70.78) | -1777.48* (-2620.69, -1456.78) | 1.69* (1.50, 1.98) |
| 95th percentile | Intercept | 241.79* (203.93, 310.07) | 5277.8* (4146.92, 6502) | -0.30* (-0.38, -0.15) |
|  | SDI | -231.22* (-307.06, -169.76) | -4772.84* (-5667.6, -3132.11) | 2.40* (2.23, 2.59) |

*significant at 5% level of significance
